# The Influence of Vacuum Level on the Milk Emission Curves and Udder Health of Saanen Goats Reared in Italy

**DOI:** 10.3390/ani15162432

**Published:** 2025-08-19

**Authors:** Mariagiovanna Domanico, Valentina D’Onofrio, Guglielmo Militello, Giuseppina Giacinti, Giuseppe Bitonti, Marcella Guarducci, Domenico Giontella, Silverio Grande, Maria Caria, Carlo Boselli

**Affiliations:** 1 Istituto Zooprofilattico Sperimentale del Lazio e Della Toscana “M. Aleandri”, National Reference Centre for Ovine and Caprine Milk and Dairy Products Quality (C.Re.L.D.O.C.), 00178 Rome, Italycarlo.boselli@izslt.it (C.B.); 2 Independent Researcher, via U. Giordano, 29, 01017 Tuscania, Italy; 3 Associazione Nazionale Della Pastorizia, 00187 Rome, Italy; 4 Department of Agricultural Sciences, University of Sassari, 07100 Sassari, Italy

**Keywords:** milk yield, Lactocorder^®^, milkability, somatic cells

## Abstract

The kinetics of milk release is influenced by several factors, including the milking facility, which affects the milk emission profile and quality. In dairy goats, the typical working vacuum level is 41–44 kPa; higher levels negatively impact health, quality, and milkability traits. This study, which was conducted on a commercial dairy farm located in the Latium region (central Italy), evaluates the impact of two vacuum levels (38 kPa and 42 kPa) on the milk emission profile and somatic cell content in Saanen goats. Statistical analysis (one-way ANOVA) of 400 milk flow curves recorded from 100 goats in four different afternoon milking sessions (200 at 42 kPa and 200 at 38 kPa) showed no significant differences in terms of milk yield, total milking time, or bimodal curve percentage when using two different operating vacuum levels. However, the milk emission time was longer at 38 kPa (1.86 vs. 1.71 min), while the peak flow rate (1.04 vs. 0.96 kg/min) and blind time (0.32 vs. 0.24 min) were higher at 42 kPa. Somatic cell content decreased significantly as the working vacuum level decreased (2470 vs. 2167 × 1000 cells/mL). This is in line with current studies which suggest that high vacuum levels increase the risk of udder injury and intramammary infection. In conclusion, adjusting the milking machine to a working vacuum level of 38 kPa, and performing proper maintenance and routine checks, significantly improves somatic cell content, and, consequently, milk quality, in goats.

## Simple Summary

This paper discusses how milking settings affect milk yield and quality in Saanen goats. Previous research has suggested that high vacuum levels might decrease the milk quality and udder health. The present study compares two different vacuum levels (38 and 42 kPa) by analyzing milk emission flow and somatic cell content. The results show that a lower vacuum level led to a significant reduction in somatic cells in milk. These results support the idea that setting the vacuum to a lower level (38 kPa), combined with good maintenance and a good routine, can improve milk quality and health in dairy goats.

## 1. Introduction

In dairy species, the milking routine and working parameters of the milking machine, such as the vacuum levels, frequency, and pulsation ratio, are crucial factors affecting milk yield, workload during milking, teat condition, and udder health. Due to differences in udder and teat anatomy between dairy animals, it is necessary to study species-specific milking procedures and the settings of the milking machine [1].

In general, the pulsation frequency is species-specific. It ranges from a minimum of 60 cycles per minute for cows and buffalos to 90 cycles per minute for goats and up to 150–180 cycles per minute for dairy sheep. Conversely, the operating vacuum level should be regulated based on the type of milking machine and dairy species due to their different milk emission profiles [2].

In fact, small ruminants’ mammary gland anatomy differs from that of large ruminants. Dairy goats have larger udder compartments and a cisternal-to-alveolar milk ratio of over 50% [3]. Because of this particular type of anatomy, goats can tolerate longer intervals between two milking sessions and permit different milking strategies to be employed [4].

Milkability is defined as the ability of an animal to give regular, complete, and rapid milk secretion via the mammary gland in response to a proper milking technique [5]. The milking machine’s design and parameter setting affect the milk yield, milk quality, hygiene, and milking time. Each dairy species has its own range of parameters and machine settings such as vacuum level, pulsation rate, and pulsation ratio, which may have effects on animal health and milk quality [6,7].

In fact, the fine-tuning of the milking machine through the settings of the operative parameters allows the mechanical action of the system to be adapted to the animal species, whereas the correct sizing and choice of the system components contribute to the regular milking process in terms of vacuum stability, milking times, and animal health [2]. Different studies have shown how the use of high vacuum levels entails an increase in the teat thickness variation after milking, with possible consequences for udder health in the long term, and negative effects on production quality [1,2,8].

On Italian goat farms, the working vacuum levels are generally set between 41 and 44 kPa, with only a small percentage set at 37–41 kPa [2]. Even lower values (36 kPa) were reported by Billon et al. [9].

In goat’s milk, the somatic cell count refers to the total number of somatic cells—mainly leukocytes (particularly macrophages and neutrophils), as well as exfoliated epithelial cells—per milliliter of milk [10]. The somatic cell count is an important indicator of udder health. Unlike cow’s milk, goat’s milk naturally exhibits higher SCC values, even in the absence of infection. This is due to non-infectious factors (e.g., phenotypic, reproductive, and lactation-related factors), infectious factors (bacteria and fungi are commonly susceptible to intramammary infections, as are lentiviruses), and the apocrine mechanism of milk secretion in goats [11]. This mechanism involves the release of cytoplasmic fragments from mammary epithelial cells, thus physiologically increasing the concentration of somatic cells [4]. Although an increase in SCCs is associated with multifactorial causes, it can also be linked to incorrect settings for key milking machine parameters, particularly the vacuum level [1,12].

Milk yield and milkability can be observed through the milk flow profiles recorded by a portable electronic milk meter, e.g., the Lactocorder^®^ (WMB, Balgach, Switzerland), usually used to record milk yield, electrical conductivity, and milk flow curve parameters [5].

This study aims to estimate the impact of two different working vacuum levels on the milk yield, milkability, and udder health (SCC) of Saanen goats reared on a commercial farm located in the Latium region of Italy.

## 2. Materials and Methods

The study was carried out on a commercial dairy farm for goats located in the Latium region of Italy over a period of two weeks (Figure S1).

At the time of the study, the herd consisted of 292 lactating Saanen goats kept in a semi-intensive system and fed with concentrate, alfalfa hay, polyphyte grass, and dehydrated beet pulps. A total of 100 goats, with parity 2.7 ± 1.2 and 58 ± 34 days in milk at the start of the trial, were monitored using electronic milk meters during the afternoon milking sessions in the first two weeks of April. The vacuum level of the milking machine was set at 42 kPa in the first week and at 38 kPa in the second week.

A standard pre-milking routine was adopted, including teat cleaning, a short massage, and drying.

The milking routine was performed by a single milker. The milking cluster was attached to the teats after the pre-milking routine and was then removed manually by the same milker at the end of the mechanical milking process.

The milking system with a low line (DeLaval, Tumba, Sweden) was designed with 12 milking stalls and set at 90 cycles per minute with a 60% ratio.

The clusters were equipped with liners featuring a mouthpiece lip diameter and an overall length of 20–185 mm.

Milk flow curves (100 curves/milking session) were recorded at random during evening milking for 2 days at 42 kPa and 2 days at 38 kPa using 3 electronic mobile milk flow meters (LactoCorder^®^, WMB, Balgach, Switzerland) [13,14,15,16,17].

The electronic Lactocorder^®^ milk meters were vertically attached to every milking place and inserted between the milking unit and milk pipeline via a milk hose in the long milk tube. The Lactocorder^®^ started to record and store information as soon as the first teatcup was attached and identification of milk flow traits was performed by the LactoPro software (Version 5.2.0; WMB, Balgach, Switzerland).

During each milking session, the Lactocorder^®^ device automatically collected representative individual milk samples, which were then refrigerated (4 °C) and transferred to the milk laboratory of the Istituto Zooprofilattico Sperimentale del Lazio e della Toscana “M. Aleandri” (IZSLT, Rome, Italy). This laboratory is accredited by Accredia (the Italian Accreditation Body, laboratory number 0201A) and adheres to the requirements of the International Organization for Standardization (ISO/IEC 17025:2017) [18].

The SCC (cell/mL) analysis was performed within 24 h of collection by a Fossomatic FC fluoro-optometric device (Foss Electric, Hillerød, Denmark) using a fluoro-optometric method. The device was calibrated using appropriate milk goat standards (low-, medium-, and high-level Goat Milk–Special Cell Count Standards), obtained from the Milk & Dairy Institute Dr. Hüfner (laboratory Dr. Hüfner GmbH, Hergatz, Germany).

The following is a list and a brief description of the information collected by the Lactocorder^®^ in this study:

- Milk Yield (MY, kg): total milk yield from the beginning to the end of the mechanical milking.
- Peak Flow Rate (PFR, kg/min): milk flow rate when the majority of milk is released.
- Milk Emission Time (MET, min): the time between having a milk flow rate of over 0.50 kg/min and having a milk flow rate of 0.20 kg/min.
- Plateau Phase Time (PPT, min): the length of the phase with constant milk flow.
- Time of Decline Phase (DPT, min): length of the decline phase, from the end of the PPT to a milk flow of 0.20 kg/min.
- Average Flow Rate (AFR, kg/min): average milk flow rate during the MET.
- Blind Time (BT, min): mechanical overmilking recorded from the end of the DPT to a milk flow below 0.10 kg/min.
- Total Milking Time (TMT, min): the time elapsed between the attachment of the milking cluster to the manual detachment at the end of milking.
- Bimodality (BIMO): milk flow curves with delayed milk ejection at the beginning of milking due to the interruption or reduction of milk flow at the beginning of milking.

Statistical analysis: To evaluate the effect of the two different working vacuum combinations on milk yield, milkability parameters, and SCC, the data and variance, with Bonferroni adjustment, were analyzed through one-way ANOVA in MedCalc^®^ Statistical Software version 23.1.3 (MedCalc Software Ltd., Ostend, Belgium; https://www.medcalc.org; accessed on 17 August 2025). Differences in least-squares means were tested with the Scheffé post hoc test at *p* < 0.05. A chi-squared test was used to analyze differences in the percentage of bimodal curves between the two tested vacuum levels (38 kPa and 42 kPa). Results are presented as mean ± SD.

## 3. Results and Discussion

The descriptive statistics of MY, milkability traits, and SCC are summarized in Table 1.

The MY mean result was 1.24 ± 0.48 kg for each sampled goat, while the PFR and AFR were 1.01 and 0.67 kg/min, respectively, similar to those values reported by other authors for this breed [19,20] but lower than those for Alpine goats [21]. On average, the MET accounted for 75.1% of the TMT (ratio of mean). The SCC was found to be 2318 ± 1497 (×1000, cell/mL), which is consistent with results reported by other researchers for this breed [11,12]. In the present study, the bimodality of the milk flow curves was found to be 16.6%, which is higher than the 6.78% recorded for Saanen goats by Šlyžienė et al. [19].

To different degrees, both the working vacuum levels affected the milk flow parameters and SCC. The collected data are summarized in Table 2.

The MY was greater when using the lower vacuum level, with a milk production of 1.27 kg for 38 kPa and 1.22 kg for 42 kPa. The MET was significantly longer in the milk flow curves recorded at the low vacuum level (1.86 vs. 1.71 min), while the PFR (0.96 vs. 1.04 kg/min) and the BT (0.24 vs. 0.32 min) were higher in the milk flow curves recorded at the highest vacuum level.

The PPT and DPT were different between the two vacuum levels. In fact, the PPT was significantly longer at 38 kPa (0.97 min) compared to at 42 kPa (0.59 min), while the DPT was shorter at 38 kPa (0.66 min) compared to at 42 kPa (0.78 min). The AFR was lower at 38 kPa (0.65 kg/min) compared to at 42 kPa (0.68 kg/min). In addition, the TMT was similar for both vacuum levels tested, with values of 2.47 and 2.33 min for 38 and 42 kPa, respectively.

The percentage of bimodal curves showed no significant variation between the two vacuum levels (14.5% at 38 kPa vs. 18.5% at 42 kPa). The SCC values were 2167 (×1000 cell/mL) for 38 kPa and 2470 (×1000 cell/mL) for 42 kPa, decreasing significantly at the lower vacuum level.

Our data show how the application of different working vacuum levels (38 kPa and 42 kPa) to the teats during the mechanical milking affected the milkability and the udder health in the Saanen goats enrolled for this study. There are different studies that show how the use of high vacuum levels entails an increase in the teat thickness after milking, with possible consequences for udder health in the long term, and negative effects on production quality [1,2,8,22].

The MY mean (1.24 kg) for each sampled goat was close to the values reported in the literature for this breed: 1.33 ± 0.07 and 1.15 ± 0.51 kg per milking [19,23]. The PFR and AFR mean were 1.01 and 0.67 kg/min, respectively. These values are similar to those reported by other authors for this breed: 0.94 ± 0.04 and 0.70 ± 0.03 kg/min [11] and 1.01 ± 0.44 and 0.71 ± 0.30 kg/min [23]. However, they are lower than those reported for Alpina goats: 1.05 ± 0.34 and 0.75 ± 0.22 kg/min, respectively [21].

The MY did not show significant statistical differences between the two working vacuum levels, with a production of 1.27 kg of milk at the 38 kPa vacuum level and 1.22 kg at 42 kPa. These data are consistent with the findings of other authors who did not observe different production when using different pressure vacuum levels [1,2], even when the studies were conducted on the Alpine breed.

The AFR, too, was not significantly statistically different between the two vacuum levels applied, with a production of 0.65 kg/min for 38 kPa and 0.68 kg/min for 42 kPa. Similar data were found in studies conducted on Alpine breed goats [2].

The TMT mean was 2.47 min at 38 kPa and 2.33 min at 42 kPa. As expected, the milking time was shorter at the higher vacuum level; however, the difference of 0.13 min was very small.

Instead, the MET was statistically significant between the two parameters, showing a longer time at 38 kPa (1.86 min–111.6 s) than at 42 kPa (1.71 min–102.6 s). This is in accordance with the findings of other authors who found a decreased milking time at higher vacuum levels [1,2,8].

The PFR and BT were lower at 38 kPa (0.96 kg/min and 0.24 min) than at 42 kPa (1.04 kg/min and 0.32 min), and these data results are statistically significant. This was expected due to the lower pressure applied by the machine to the organ. Similar data were reported in the literature by another author [2]. The PPT was significantly longer at 38 kPa (0.97 min) compared to at 42 kPa (0.59 min), and may be related to oxytocin taking longer to empty the alveolar milk fraction. These findings are in accordance with what was found by Caria et al. [2]. Although it is well established that dairy goats store a large percentage of their milk in the glandular cistern and, after milking cluster attachment, milk is available from the beginning of the milking process, oxytocin release is mandatory to obtain the maximum yield and fat from the udder, minimizing the amount of residual milk. Furthermore, this oxytocin release produces a second peak in the milk flow, and, if the oxytocin release occurs while the cisternal fraction has not been completely removed, the aforementioned second peak in the milk flow occurs even earlier and the two peaks overlap, thus increasing milking efficiency [24]. In the milk flow curves, this phenomenon can be observed without a clear separation between the plateau and the decreasing milk flow phase.

Instead, the DPT was longer at the low vacuum level (0.66 min) than at the high one (0.78 min), being always correlated with the lower pressure applied to the udder by the milking machine.

Goats have a larger cisternal portion than cows which fills earlier than the alveolar fraction and is the first to empty during milking; in fact, the ejection of the alveolar milk occurs after stimulation by oxytocin [25,26]. Due to this udder anatomy and arrangement of the mammary tissue, the cisternal fraction of milk is ejected before the alveolar fraction, often giving a long period of vacuuming without any or low ejection of milk and, consequently, a bimodal milk flow curve [20]. Long delays may indicate suboptimal pre-stimulation of the teats or insufficient oxytocin release in the bloodstream, resulting in undesired protracted interruptions between the two milk peaks [27]. Although the prevalence of bimodal curves was high for both vacuum levels tested, no statistically significant differences were found in our study. In fact, 14.5% of the curves were classified as bimodal at 38 kPa compared to 18.5% at 42 kPa.

The somatic cell count is considered an indirect indicator of milk hygiene. Higher SCC values, above the normal physiological range, suggest microbial inflammation of the mammary gland. However, this is not entirely applicable to goat’s milk, as it naturally contains higher levels of epithelial cells and their fragments due to epithelial desquamation and the natural regeneration of mammary alveoli [28]. In our study, the SCC was lower when using the 38 kPa vacuum (2167 × 1000 cells/mL) than the 42 kPa vacuum (2470 × 1000 cells/mL), with a statistically significant difference.

Authors have reported for small ruminants a higher SCC and an increase in the thickness of teat end tissue (>5%) when using a 44 kPa vacuum [8]. On the contrary, Romero et al. [29], in a field study conducted on Murciano-Granadina goat breeds, found, by ultrasound examination of the teats, that an increase in vacuum level (from 36 and 42 kPa) corresponded to a significant increase in teat wall thickness, teat wall area, and teat end wall area, without an increase in the somatic cell count in the milk.

Sinapis et al. [30] found a higher probability of occurrence of mastitis infections due to traumatic lesions to teat ends at high vacuum levels (>38 kPa). They also found a difference in the log SCC (*p* < 0.05) between 38 and 50 kPa. A vacuum level of 36 kPa seems to be less stressful for teat tissue and more efficient in terms of milking performance than the extreme vacuum levels [1].

On the other hand, Romero et al. [29], regarding the relationship between vacuum level and udder health, in an experiment conducted on Murciana-Granadina goats, observed that using intermediate vacuum values (such as 38 kPa) when milking with a low-speed machine achieves a balance between milking efficiency and animal health. Nevertheless, overmilking, visible in milk flow curves through the BT, applied during the whole lactation process, along with the increase in the teat thickness, may contribute to the development of new intramammary infections, and, consequently, an increase in SCC [4]. The same study [4] also found that overmilking has a more pronounced effect on teat tissue in goats than in sheep. Olechnowicz [31] reports that long overmilking times with inadequate vacuum levels and an inadequate pulsation ratio can damage teat ends and cause injury. This allows microorganisms to penetrate the mammary gland, causing inflammation and microbial contamination of the milk. This affects the somatic cell number and composition and milk production.

Furthermore, we observed a non-significant decrease in bimodal curves (*p* = 0.58) when passing from 42 kPa to 38 kPa. Šlyžienė et al. [19] indicated that bimodality in goat’s milk is associated with an increase in log_10_ SCC (*p* < 0.05). They also support the hypothesis that bimodality has an adverse effect on the health of goats and should therefore be avoided.

Incorrect application of operative parameters such as vacuum level and milking machine components may affect milking performance, animal health, and, consequently, milk quality. In general, misapplication of operational parameters, such as vacuum level and milking machine components, can affect milking performance and animal health, consequently impacting milk quality. It is important to emphasize that improper maintenance of the milking machine, particularly the use of incorrect liner types and operational parameters, can affect the performance of mechanical milking, such as milking time and milk yield [2].

Additionally, the condition of the teatcups in the milking cluster is critical for ensuring udder health, especially when combined with inappropriate vacuum levels during milking. Michael et al. [32] observed this phenomenon in a field study conducted in Greece on 321 small ruminant farms (255 sheep and 66 goat farms). Among these, 150 farms showed irregular teatcup conditions (such as tears or cracks), leading the authors to conclude that the risk of mastitis significantly increased when milking vacuum levels exceeded 42 kPa [32].

A proper milking machine accommodates all teat sizes and forms and has a low vacuum to effectively open the teat and to stimulate physiological milk release and letdown [22].

Our study therefore agrees with what is reported in the literature, showing how proper maintenance of the milking machine, including the state of wear of the teatcups, the application of a vacuum level of 38 kPa, and an adequate routine of milking techniques contribute to the reduction of the SCC level without lengthening milking times. This vacuum level is consistent with the optimal milking settings recommended by Dzidic et al. [4] for goats, which include a vacuum level of approximately 38 kPa and a pulsation rate of 100–120 cycles per minute.

The milk flow curves recorded under our experimental conditions at two different vacuum levels (38 and 42 kPa) show mechanical milking times very similar to each other, with small non-significant differences.

## 4. Conclusions

Our field study confirms that the vacuum level applied during afternoon goat milking affects milkability traits and somatic cell count, while it does not seem to affect milk yield.

Our results, obtained from a commercial farm and in the short term, are consistent with previous reports by other authors on the positive impact of reducing vacuum levels during milking on mammary gland health, thereby mitigating the risk of physical stress and mastitis.

In conclusion, lower vacuum levels, particularly around 38 kPa, improve performance in terms of sanitary and milkability traits without increasing total milking times. Therefore, correctly monitoring and setting milking machine parameters—including periodically checking the condition of the teatcups—can improve the efficiency of milking procedures, reduce production costs for farmers, and enhance the welfare of dairy goats.

## Figures and Tables

**Table 1:** Overall descriptive statistics of the measured traits.

Parameters	Mean	SD	95% IC	Median
MY (kg)	1.24	0.48	1.19 to 1.29	1.18
PFR (kg/min)	1.00	0.39	0.96 to 1.04	0.90
MET (min)	1.78	0.75	1.70 to 1.85	1.68
PPT (min)	0.73	0.70	0.61 to 0.75	0.61
DPT (min)	0.70	0.57	0.66 to 0.80	0.56
AFR (kg/min)	0.67	0.23	0.64 to 0.75	0.63
BT (min)	0.25	0.33	0.21 to 0.28	0.14
TMT (min)	2.37	0.82	2.29 to 2.45	2.24
SCC (×1000 cell/mL)	2318	1497	2171 to 2466	1975
BIMO (%)	16.6	-	-	-

MY = milk yield; PFR = peak flow rate; MET = milk emission time; PPT = plateau phase time; DPT = time of decline phase; AFR = average flow rate; BT = blind time; TMT = total milking time; BIMO = bimodality; SCC = somatic cell count.

**Table 2:** Effect of two vacuum levels (38 and 42 kPa) on milk yield, milkability traits, and somatic cell count.

Recorded Parameters	38 kPa	42 kPa	*p* Value
MY (kg)	1.27 ± 0.45	1.22 ± 0.51	0.307
PFR (kg/min)	0.96 ± 0.34 ^b^	1.04 ± 0.39 ^a^	0.035
MET (min)	1.86 ± 0.73 ^a^	1.71 ± 0.76 ^b^	0.047
PPT (min)	0.97 ± 0.72 ^a^	0.59 ± 0.04 ^b^	<0.001
DPT (min)	0.66 ± 0.47 ^b^	0.78 ± 0.59 ^a^	0.030
AFR (kg/min)	0.65 ± 0.20	0.68 ± 0.23	0.268
BT (min)	0.24 ± 0.26 ^b^	0.32 ± 0.43 ^a^	0.026
TMT (min)	2.47 ± 0.74	2.33 ± 0.92	0.124
SCC (cell/mL)	2167 ± 1456 ^b^	2470 ± 1524 ^a^	0.043
BIMO (%)	14.5	18.5	0.281

MY = milk yield; PFR = peak flow rate; MET = milk emission time; PPT = plateau phase time; DPT = time of decline phase; AFR = average flow rate; BT = blind time; TMT = total milking time; SCC = somatic cell count. A chi-square test was used to compare BIMO (bimodality). Values with ^a, b^ letters are significantly different (*p* < 0.05).

## Data Availability

The original contributions presented in this study are included in the article/Supplementary Material. Further inquiries can be directed to the corresponding author.

## Supplementary Materials

The following supporting information can be downloaded at https://www.mdpi.com/article/10.3390/ani15162432/s1, Figure S1: Map of the Latium region (Italy), showing the location of goat farms categorized by the consistency class of the animals raised.

## Abbreviations

The following abbreviations are used in this manuscript:

SCC	Somatic Cell Count
MY	Milk Yield
PFR	Peak Flow Rate
MET	Milk Emission Time
PPT	Plateau Phase Time
DPT	Time of Decline Phase
AFR	Average Flow Rate
BT	Blind Time
TMT	Total Milking Time
BIMO	Bimodality
