# Supplementary material for: The Influence of Vacuum Level on the Milk Emission Curves and Udder Health of Saanen Goats Reared in Italy
Source: Animals (Basel). 2025 Aug 19;15(16):2432. doi: 10.3390/ani15162432 (PMC12382765; doi:10.3390/ani15162432)
Supplement: Supplementary file 1 [file animals-15-02432-s001.zip › animals-3759502-supplementary.pdf]

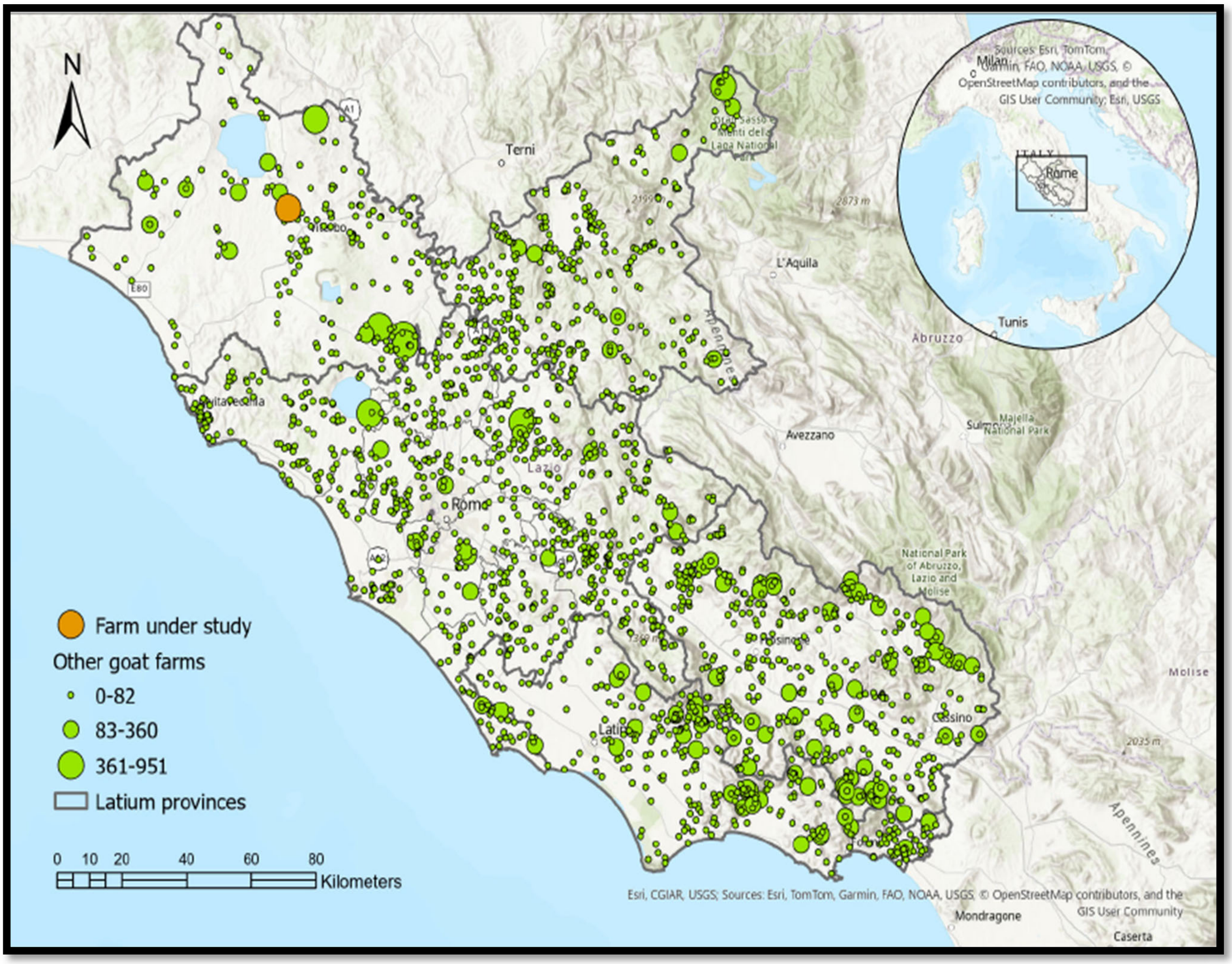

Figure S1. Map of the Latium region (Italy), showing the location of goat farms categorised by the consistency class of the animals raised.
